# The Effectiveness of Self-Management of Hypertension in Adults Using Mobile Health: Systematic Review and Meta-Analysis

**DOI:** 10.2196/17776

**Published:** 2020-03-27

**Authors:** Ran Li, Ning Liang, Fanlong Bu, Therese Hesketh

**Affiliations:** 1 Center of Global Health, School of Public Health, School of Medicine Zhejiang University Hangzhou China; 2 Institute of Basic Research in Clinical Medicine China Academy of Chinese Medical Sciences Beijing China; 3 Center for Evidence-Based Chinese Medicine Beijing University of Chinese Medicine Beijing China; 4 Institute of Global Health University College London London United Kingdom

**Keywords:** hypertension, self-management, mHealth, medication adherence, mobile phone, health behavior

## Abstract

**Background:**

Effective treatment of hypertension requires careful self-management. With the ongoing development of mobile technologies and the scarcity of health care resources, mobile health (mHealth)–based self-management has become a useful treatment for hypertension, and its effectiveness has been assessed in many trials. However, there is a paucity of comprehensive summaries of the studies using both qualitative and quantitative methods.

**Objective:**

This systematic review aimed to measure the effectiveness of mHealth in improving the self-management of hypertension for adults. The outcome measures were blood pressure (BP), BP control, medication adherence, self-management behavior, and costs.

**Methods:**

A systematic search was conducted using 5 electronic databases. The snowballing method was used to scan the reference lists of relevant studies. Only peer-reviewed randomized controlled trials (RCTs) published between January 2010 and September 2019 were included. Data extraction and quality assessment were performed by 3 researchers independently, adhering to the validation guideline and checklist. Both a meta-analysis and a narrative synthesis were carried out.

**Results:**

A total of 24 studies with 8933 participants were included. Of these, 23 studies reported the clinical outcome of BP, 12 of these provided systolic blood pressure (SBP) and diastolic blood pressure (DBP) data, and 16 articles focused on change in self-management behavior and medication adherence. All 24 studies were included in the narrative synthesis. According to the meta-analysis, a greater reduction in both SBP and DBP was observed in the mHealth intervention groups compared with control groups, −3.78 mm Hg (*P*<.001; 95% CI −4.67 to −2.89) and −1.57 mm Hg (*P*<.001; 95% CI −2.28 to −0.86), respectively. Subgroup analyses showed consistent reductions in SBP and DBP across different frequencies of reminders, interactive patterns, intervention functions, and study duration subgroups. A total of 16 studies reported better medication adherence and behavioral change in the intervention groups, while 8 showed no significant change. Six studies included an economic evaluation, which drew inconsistent conclusions. However, potentially long-term financial benefits were mentioned in all economic evaluations. All studies were assessed to be at high risk of bias.

**Conclusions:**

This review found that mHealth self-management interventions were effective in BP control. The outcomes of this review showed improvements in self-management behavior and medication adherence. The most successful mHealth intervention combined the feature of tailored messages, interactive communication, and multifaceted functions. Further research with longer duration and cultural adaptation is necessary. With increasing disease burden from hypertension globally, mHealth offers a potentially effective method for self-management and control of BP. mHealth can be easily integrated into existing health care systems.

**Trial Registration:**

PROSPERO CRD42019152062; https://www.crd.york.ac.uk/prospero/display_record.php?RecordID=152062

## Introduction

### Background

Hypertension is the underlying cause of around 20% of deaths globally [1]. The causes of hypertension are largely lifestyle related [2]. Poor control is known to be related to failure to diagnose cases and inadequate treatment [3]. On average, in high-income countries, around 67% of hypertension sufferers are diagnosed, with 55% treated and 28% achieving control. In contrast, in low- and middle-income countries (LMICs), around 37% of the hypertensives are diagnosed, of whom 29% are treated, with 8% achieving control [4]. The annual worldwide costs to the economy of hypertension are estimated at US $370 billion [5]. Considering unequal health care resource coverage, mobile health (mHealth) presents exciting potential in developing self-management for patients with hypertension with uncontrolled blood pressure (BP) [6].

### Self-Management

The World Health Organization (WHO) defines self-management as the capability of individuals to manage their own health conditions, with or without the support of health care providers [7]. The methods of self-management include education for patients, self-monitoring of clinical data, and behavior (eg, diet, exercise, smoking, and drinking), self-titration of medical management, and support for medication adherence as per prescribed regimes [8]. A systematic review conducted by Barlow et al [9] documented that education on self-management can not only improve patients’ knowledge of hypertension but can also allow for early detection of high BP. Another study reported that the self-management of hypertension enables patients to correctly self-titrate medications and irreversibly change the dynamics between doctors and patients [10]. mHealth is emerging as a vital platform to perform self-management, especially for chronic diseases [11].

### Mobile Health

mHealth is the use of mobile devices—such as mobile phones, patient-monitoring devices, and wireless devices—for medical support and the delivery of health management [12]. The use of mobile devices has increased exponentially, worldwide—over 7 billion mobile device subscriptions were reported in 2015 [13]. This clearly facilitates the feasibility, generalizability, and replicability of mHealth. WHO first defined the term mHealth in 2010. Since then, mHealth technology has been more widely available and sophisticated [14,15]. mHealth employs a variety of different features, including SMS text messages, emails, phone calls, and mobile phone apps [16]. The benefits of mHealth are widely acknowledged. It can contribute to achieving universal health coverage by overcoming geographical barriers, increasing access, and the provision of health services to remote populations and underserved communities. Clinical data monitoring and educational information communications, between physicians and patients, cost less than in-person services, as the implementation of mHealth does not utilize any further resources [17]. mHealth stands at the crossroads of communication technologies and personalized health care [18].

### Existing Research

Most existing systematic reviews have assessed the effectiveness of clinical outcomes of mHealth self-management in noncommunicable diseases, mainly diabetes, cardiovascular disease (CVD), and heart failure [19]. A few others have examined the content of the intervention, the study population, and economic evaluation [20,21]. Further research has investigated the effects of mHealth self-management intervention in relation to the adherence and self-titration of medication of diabetes [22]. Results were consistent in that mHealth-enabled self-management solutions could provide benefits for chronic conditions, increasing access to health care, as well as improving health care quality and patient involvement [23].

### Research Gap

There is limited literature looking at the effectiveness of mHealth in hypertension self-management. A systematic review narratively synthesized the evidence for using mHealth devices to support hypertension self-management. The study reported lower systolic blood pressure (SBP) and diastolic blood pressure (DBP) in the intervention group compared with usual care [24]. Only using quantitative methods, a meta-analysis of 11 randomized controlled trials (RCTs) conducted by Lu et al [25] concluded that mHealth is an effective tool for BP control. We know of no other reviews that examined the relationship between long-term self-management and cost-effectiveness. Therefore, further exploration of the relationship between mHealth-enabled hypertension self-management and clinical outcomes needs to be conducted. This would enable relevant stakeholders to weigh the potential benefits and limitations of adopting mHealth into health care services. Thus, we aimed to determine whether mHealth is effective in improving the self-management of hypertension for adults. We systematically reviewed the existing evidence to analyze the effectiveness of mHealth-enabled self-management among hypertensive adults with the following 3 objectives:

1. To measure whether the use of mHealth-enabled self-management improves the control of BP among patients with hypertension.
2. To assess whether self-management education of hypertension delivered by mHealth interventions improves medication adherence and promotes lifestyle change.
3. To analyze the costs of self-management support for the delivery of mHealth interventions for hypertension in adults.

## Methods

### Study Design

This systematic review and meta-analysis were conducted based on the original protocol (CRD42019152062) and reported according to the Preferred Reporting Items for Systematic Reviews and Meta-Analyses guideline [26].

### Search Strategy

An electronic database search was conducted using PubMed, EMBASE, Web of Science, Cochrane, and Google Scholar. Searches were performed in October 2019. The key search strings consisted of 3 concepts: mHealth, hypertension, and self-management. The detailed search strategy has been presented in Textbox 1.

**An example of the search strategy (EMBASE).**

- Search 1: (hypertension* or hypotension or hypertensive or “blood pressure”* or “elevated blood pressure” or “high blood pressure'”).af.
- Search 2: (self-management* or “self care”* or “self management” or “self monitoring” or self-monitoring or self-care).af.
- Search 3: (telemedicine* or telehealth or eHealth* or “e health” or e-health or mHealth* or “m health” or m-health or “mobile application” or apps or “digital health” or “mobile health” or “message text”).af.
- Search 4: limit year = “2010 - 2019”
- Search 5: 1 and 2 and 3 and 4

### Eligibility Criteria

We included RCTs published between January 2010 and September 2019. Only peer-reviewed articles in English were included.

Inclusion criteria were as follows: (1) adults with a primary diagnosis of hypertension; (2) the intervention used app-based tools that are accessible via mobile phone or tablet to aid self-management of hypertension; (3) reported outcomes were any one of the following: clinical data for either SBP or DBP or both, medication adherence-related outcome or change in self-management behavior.

The exclusion criteria were as follows: (1) patients with hypertension were not the main participants; (2) hypertension during pregnancy; (3) hypertension not the primary diagnosis; (4) the intervention only targeted health care providers; (5) outcomes only focused on technological development of a mobile system; (6) the mHealth service was designed for a particular target audience instead of the general public.

Observational studies, study protocols and designs, studies with abstract presentations, and duplicates were all excluded.

### Study Selection

Study citations were imported and compiled into the reference management software (Endnote X8.0, Clarivate Analytics) for selection. The screening and selection of studies were conducted by 3 researchers individually (RL, NL, and FB). RL manually removed duplicates. For the initial search, RL and FB independently judged the relevance of titles and abstracts identified from electronic databases. In the second phase, FB and NL checked the study types of all remaining studies. The full text of potentially relevant articles was then retrieved. NL and RL assessed these articles against the inclusion and exclusion criteria. The snowballing method was conducted on the reference lists of relevant articles. Controversial studies and problems were compared and discussed with RL, FB, and NL.

### Data Extraction

Three investigators (RL, NL, and FB) in parallel extracted the data independently and cross-checked. An adapted version of a standardized spreadsheet was used to input the data. Any disagreements were resolved through discussion. The data included the study characteristics (title, authors, year of publication, and study location); information of participants (age, gender, baseline BP, demographic information, and sample size); details about intervention and control (device, intervention and control type, message content, follow-up duration); and relevant outcome and result.

### Data Synthesis and Analysis

The primary outcomes of this review were the mean SBP, DBP, and the proportion of subjects with controlled BP at the end of each trial. Patients with hypertension with SBP lower than 140 mm Hg and DBP lower than 90 mm Hg were considered to have adequate BP control [27]. Review Manager of the Cochrane Collaboration (RevMan 5.3, Cochrane Organization) was used to perform the meta-analysis.

For continuous outcomes, the effect size was defined as the mean differences (MDs) in BP between intervention and control groups. For dichotomous outcomes, the effect size was defined as the odds ratio (OR) of the proportion of patients with controlled BP between intervention and control groups. OR and MDs were derived from Manzel-Haenszel and inverse variance methods, respectively. A random-effect model was utilized to generate pooled estimates of the overall effects and reported 95% confidence intervals with all measures of effect. The *I*^2^ statistic was used to examine inconsistencies across studies (*I*^2^=0%-100%; more than 50% is considered as substantial statistical heterogeneity). We defined subgroups in advance to further evaluate the relationship between intervention characteristic and the clinical effect on SBP and DBP controlling for (i) frequency of reminders (tailored frequency according to the health status of participants or fixed frequency as planned); (ii) interactive patterns (interventions with a patient-provider loop interaction or without); (iii) intervention functions (single and multifaceted); and (iv) duration of trials (longer or equal to 12 months or shorter than 12 months). A sensitivity analysis was performed by excluding each study sequentially to determine the influence of any single study on the robustness of the results.

Owing to the heterogeneity in the nature of interventions and diverse outcomes of effects of self-management, we have also presented a narrative review of the findings, including structured tabular summaries according to medication adherence, change in self-management behavior, and costs.

### Assessment of Risk of Bias and Quality

To account for bias and quality discrepancies, a double assessment of bias and quality was used to minimize error. RL, FB, and NL independently assessed the risk of bias and quality of the included studies. A consensus meeting was held to enable the comparison of notes from the selection of papers used within this review. An agreement was reached on conflicting points.

The risk of bias was assessed according to guidance in the *Cochrane Collaboration’s Risk of Bias* handbook for RCTs [28]. Risk ratings of “low,” “high,” and “unclear” were assigned to each bias based on the presence of the following items: selection bias, performance bias, detection bias, attrition bias, reporting bias, and other bias. If any element was rated as high risk, the overall risk of bias was high. Funnel plots were used to detect publication bias if over 10 articles were involved in the meta-analysis. When the description of the interventions or procedures was not sufficiently detailed to judge the risk of bias, researchers contacted the study author for further information.

The quality of the included studies was evaluated by the Mobile Health Evidence Reporting and Assessment (mERA) Checklist [29]. This checklist was developed from the Consolidated Standards of Reporting Trials of Electronic and Mobile HEalth Applications and onLine TeleHealth (CONSORT-EHEALTH) checklist and has been extended to a wide range of mHealth interventions. It consists of 2 separate assessments: (1) essential criteria for mHealth, which comprise 16 items that identify the content, context, and technical features, to ensure the quality and generalizability of mHealth research. (2) methodological criteria, which include 26 items that are based on existing study design and study-reporting guidelines. The result was displayed by using the rate of reported items.

## Results

### Search Results

**Figure 1:**
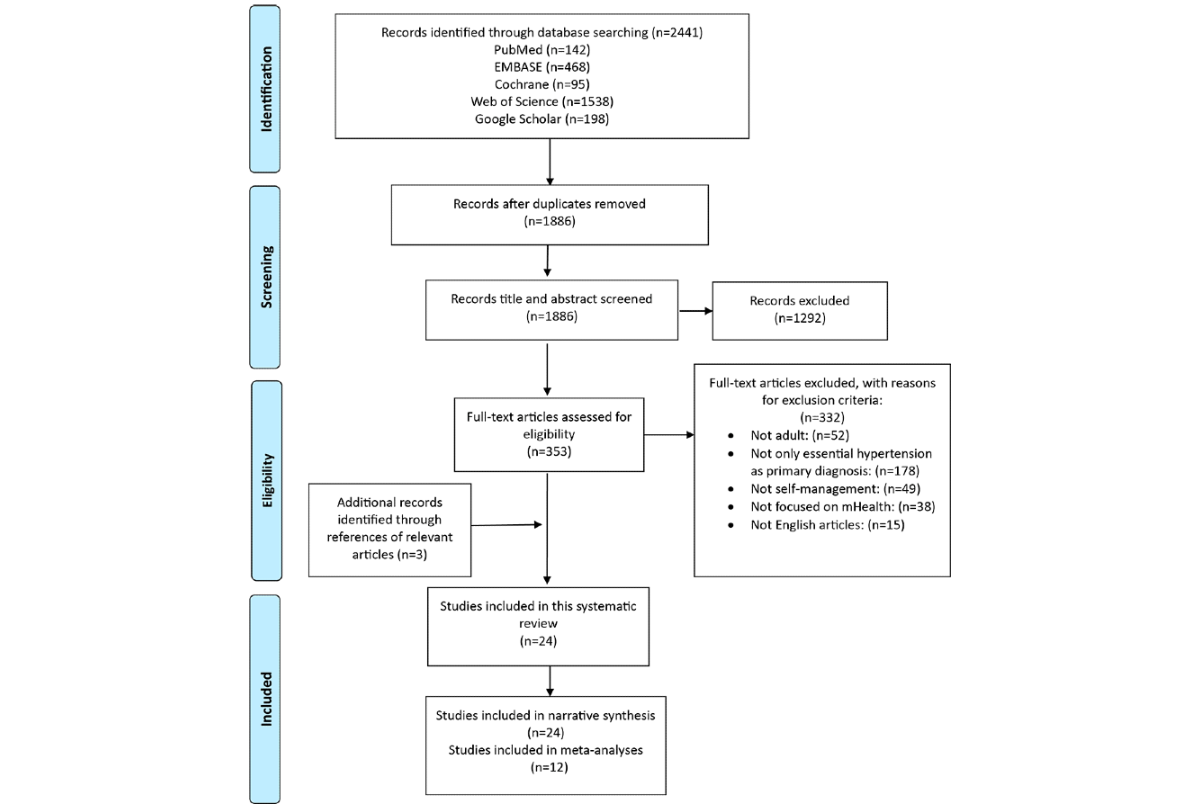
Flow diagram of study selection. mHealth: mobile health.

A total of 2441 articles were initially identified from the 5 databases; 555 articles were removed because of duplication. The remaining 1886 studies were then screened. We excluded 1292 articles because of insufficient relevance of the title and abstract to this review. A further 232 articles were rejected because they were not RCTs. A total of 9 studies were excluded as only the abstracts of protocols were available. After reviewing the full text of the remaining 353 articles, 332 studies were eliminated following the application of inclusion and exclusion criteria. An additional 3 articles were identified from references of relevant reviews, yielding a total of 24 studies. All of the 24 studies were included in narrative synthesis. Of these, 12 studies that provided SBP and DBP data and used usual care or placebo as comparisons were included in the meta-analysis. A flow diagram of the selected studies is shown in Figure 1.

### Study Characteristics

Characteristics of the 24 studies are shown in Multimedia Appendix 1. Of which, 3 studies were from the United Kingdom, 11 from the United States, 3 from Canada, and 1 each from Spain, Taiwan (China), Chile, South Africa, Mexico, Iran, and South Korea.

Two studies were conducted in rural areas [30,31] and 22 in urban areas [32-53]. Four articles specifically targeted ethnic minorities and underserved populations [32,44]. The age range of patients across the 24 articles was 44 to 78 years. The duration of the trials ranged from 1.5 to 18 months. Half of the projects lasted for no more than 6 months. The mean sample size was 372, with a range from 54 to 1372, and 13 articles reported missing data during follow-up [32,35-46].

In all, 18 studies drew on existing theories or models to guide the intervention design [30,32,34-38,40,41,43-45,47-52]. JNC7 Guidelines and National Institute for Health and Care Excellence guidelines were most frequently used for hypertension treatment. Among 12 articles that were built on behavioral change theories [30,31,35,36,41,42,45-50], the social cognitive theory and determination theory were adopted the most. All 18 studies used treatment as usual (TAU) as a control [30-35,37-39,42-47,49,51,52]. The remaining 6 articles used the sending of different messages to the control group compared with the intervention group [36,40,41,48,50,53].

A total of 23 studies measured BP reduction [30-52]. Of which, 13 used the change in BP as their primary outcome [31-33,37-39,41-43,47,49-51] and 5 used BP control as the main outcome [33,42,44,46,52]. A total of 12 studies reported medication adherence [32-35,37,39,41,42,44,49-51]. Another 9 studies assessed the change in effectiveness of self-management behaviors [35-38,41,43,49,53]. The outcomes of self-management behaviors were varied, including readiness for behavior change, quality of life, and action plan protocol adherence. Among the 24 studies, 6 articles reported results of economic evaluation [37,42,44,46,52]. Finally, 6 articles analyzed stakeholders’ satisfaction and experience with the intervention [31,32,35,37,47,50].

### Intervention Characteristics

To synthesize the effects of intervention features on self-management of hypertension, this review categorized the intervention content into 13 themes: educational information of hypertension, educational information of a healthy lifestyle, self-monitoring of BP, self-monitoring of behavior change, goal setting, reminder of medication adherence, reminder of behavior change, feedback from personnel, social support, motivational encouragement, action plan, pharmacological support, and stress management. Every intervention included at least two features. Education about hypertension was included in every study. A total of 17 studies conducted BP self-monitoring [30,31,33,37,38,40-46,48,50-53]. Education about a healthy lifestyle that comprises a low-salt diet and exercise combined with goal setting in 15 studies [30,31,34-37,41,45-49,51-53]. A total of 10 studies set alerts to improve medication adherence [31,33,38,39,45-47,50-52]; 4 studies provided motivational encouragement to strengthen the patient’s self-efficacy [30,43]; 3 studies provided a decision support system for an action plan made by doctors, pharmacists, or nurses [36,48,53]; and 1 intervention included stress management [49].

A total of 6 articles included more than one intervention group [32,36,43,46,49,52,53]. Of which, 3 articles targeted the differences in effectiveness between user-driven self-management and self-management with interactive supports from personnel [32,36,46], and 1 article measured different outcomes based on varied compliance [34].

### Intervention Delivery

The information about the intervention and control design was based on the intervention platform, type, and content (Multimedia Appendix 1). There were 10 interventions delivered by using SMS text messages [32,34,37,43-45,47,49-51], half of which generated automated messages and other customized messages based on the feedback of participants [32,44,45,49,50]. Six studies utilized smartphone apps [39,43,44,47,50,51] and 2 of them were interactive [47,50]. Then 6 studies reported the operation of automated emails [30,31,33,36,40,47]. Other intervention devices included wireless BP monitoring, digital medications automated or interactive voice calls, electronic medication trays, and a combination of the elements mentioned earlier.

### Timing and Frequency

Intervention frequency varied considerably, and the fidelity of the intervention was not always reported. For most of the studies, a reminder to monitor BP was sent at least once a day. Most of messages about behavior or medication adherence were sent daily, and educational emails and feedbacks were sent weekly. Adherence to the intervention protocol was reported in 10 articles [30,31,35,38,39,41-45]. Six articles reported the difference between the required frequency of mHealth use and the actual use [31,33,35,45,50]. The range was from 28% to 94%.

### Outcome Measures

Of the total 24 studies, 12 met the selection criteria of the meta-analysis [31-33,37-39,41-43,47,49,51]. All of them reported SBP as the primary outcome; 9 of them reported the DBP as well [33,37,38,41-43,47,49,51] and 9 articles reported the proportion of participants achieving controlled BP [31-33,35,39,41,42,44,49-51].

### Meta-Analysis of Blood Pressure

**Figure 2:**
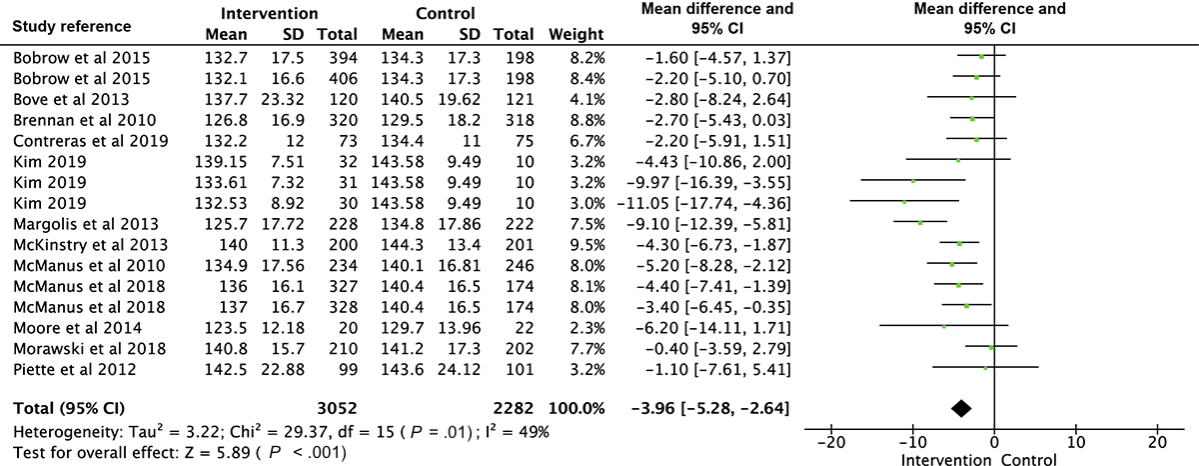
Forest plot of the difference of systolic blood pressure between intervention and control group.

**Figure 3:**
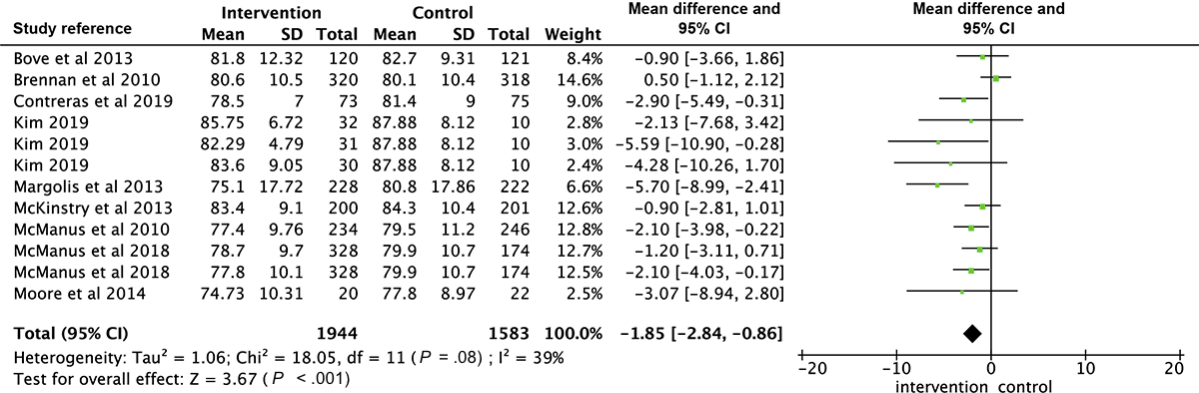
Forest plot of the difference of diastolic blood pressure between intervention and control group.

**Figure 4:**
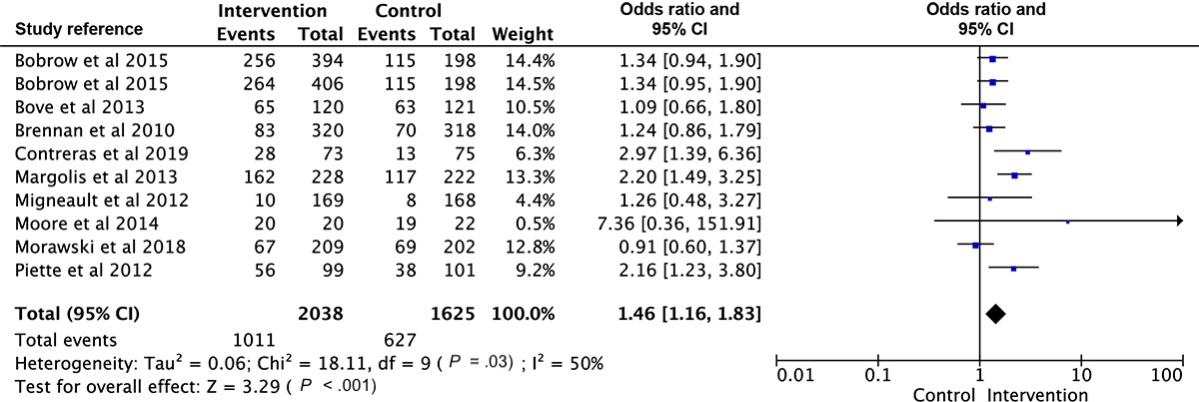
Forest plot of the difference of blood pressure control between intervention and control group.

A total of 3 articles had more than one intervention group with the same outcome measured [32,43,49]. Therefore, 16 interventions were shown in the forest plot of SBP, 12 interventions for DBP analysis, and 10 interventions for the comparison of BP control. As shown in Figure 2, the estimated MD of SBP between intervention and control groups was significant as −3.78 mm Hg (*P*<.001; 95% CI −4.67 to −2.89), with moderate heterogeneity (Χ^2^_15_=29.37, *P*=.01; *I*^2^=49%). There was a statistically significant difference in DBP as −1.57 mm Hg (*P*<.001; 95% CI −2.28 to −0.86) between intervention and control groups, also shown in the forest plot with low heterogeneity (Χ^2^_11_=18.05, *P*=.08; *I*^2^=39%; Figure 3). The OR of BP control (Figure 4) in the intervention group was 1.42 times more than that in the control group (95% CI 1.23 to 1.65). Heterogeneity was moderate (Χ^2^_9_=18.11, *P*=.03; *I*^2^=50%).

Subgroup analyses were generally consistent with the main finding, showing significant reductions in SBP and DBP in the intervention groups for all subgroups analyzed (Table 1). Trials with a tailored frequency of reminders [32,37,41,42], a patient-doctor interactive loop [31,32,38,41,42,47,49], and multifaceted functions [31,37,38,42,49] showed a larger overall effect of both SBP and DBP, compared with trials with a fixed frequency of reminders [31,33,38,39,43,47,49,51], a noninteractive loop [33,37,39,43,51], and a single function [32,33,39,41,47,51]. The overall SBP reduction is greater in studies that lasted less than 12 months [31,33,37,39,49,51] than studies that lasted longer than 12 months [32,38,41,47]. Further sensitivity analyses were conducted by removing any trials sequentially, revealing no substantial difference in the overall effect for SBP and DBP. In addition, Margolis et al [42] was regarded as the main article that influenced the heterogeneity of the comparison of SBP according to the sensitivity analysis. After excluding it from the analysis, the *I*^2^ statistic was reduced from 49% to 24% (*df*=15; *P*=.18).

**Table 1:** Results from the subgroup analyses of mean differences in systolic blood pressure and diastolic blood pressure.

Analysis			Systolic blood pressure					Diastolic blood pressure				
			Studies, n	Mean difference (mm Hg)	95% CI	Heterogeneity		Studies, n	Mean difference (mm Hg)	95% CI	Heterogeneity	
						*I*^2^ (%)	*P* value				*I*^2^ (%)	*P* value
All studies			12	−3.78	−4.67 to −2.89	49.0	.01	9	−1.57	−2.28 to −0.86	39.0	.08
**Subgroup analysis**												
	**Reminder frequency**											
		Tailored frequency	4	−4.04	−6.67 to −1.41	70.0	.009	3	−2.27	−5.03 to 0.49	70.0	.04
		Fixed frequency	8	−3.86	−5.41 to −2.30	36.0	.11	6	−1.70	−2.77 to −0.64	29.0	.19
	**Interactive pattern**											
		Interactive loop	7	−4.88	−7.00 to −2.75	61.0	.006	5	−2.71	−4.82 to −0.59	62.0	.01
		Noninteractive loop	5	−3.17	−4.47 to −1.87	0.0	.46	4	−1.54	−2.49 to −0.59	0.0	.71
	**Intervention functions**											
		Multifaceted functions	6	−5.51	−7.25 to −3.77	44.0	.08	4	−2.20	−3.27 to −1.13	23.0	.24
		Single function	6	−2.02	−3.41 to −0.63	0.0	.78	5	−0.03	−1.39 to 1.33	0.0	.40
	**Duration**											
		Shorter than 12 months	5	−4.30	−7.00 to −1.59	55.0	.04	4	−1.82	−3.06 to −0.58	0.0	.47
		Longer than 12 months (12 months included)	7	−3.89	−5.44 to −2.34	50.0	.04	5	−1.85	−3.37 to −0.33	62.0	.02

### Medication Adherence and Self-Management Behavior

This review narratively synthesized the outcome of medication adherence and self-management behavior (Multimedia Appendix 2). A total of 7 articles reported statistically significant improvement in medication adherence in intervention groups [32,34,39,41,49-51]. Five studies suggested that mHealth interventions improved medication adherence, despite nonsignificant outcomes [33,35,37,41,44]. Morisky Medication Adherence Scale was used in 6 studies [35,37,39,42,50,51]. As a result, Bove et al [33] reported that there is no association between medication adherence and BP control, that is, an improvement in adherence did not necessarily lead to better BP control. Of the 9 articles that focused on the behavioral change of self-management, all reported positive effects either through physical activities or through a healthier diet. Adverse events reported in studies, such as medication side-effect and cardiovascular event, were unrelated to self-management and were evenly distributed across intervention and control groups. An exception to this was McKinstry et al [37], who found 3 patients became anxious as a result of self-monitoring. Of which, 6 studies conducted qualitative research about satisfaction related to the intervention [31,32,35,37,47,50]. All showed high levels of satisfaction. Patients and physicians were keen continuing to practice mHealth.

### Economic Evaluation

A total of 6 articles measured the cost of mHealth (Multimedia Appendix 2) [37,42,44,46,52]. In cost-saving analyses, 2 reported that the cost of mHealth interventions was higher than control [37,42]. Two studies found the cost of mHealth interventions was lower than that of control [44,47]. The measurements of expenditure varied between study settings. The main cost was from monitoring, mobile phone use, connection charges, and cost of nurse support. However, Davidson et al [44] adjusted the BP control effect into the cost analysis, which means that patients with controlled BP after receiving the experimental treatment saved the extra cost of further treatment. This showed an overall health care cost saving of over US $20,000 between intervention and control groups.

### Risk of Bias

**Figure 5:**
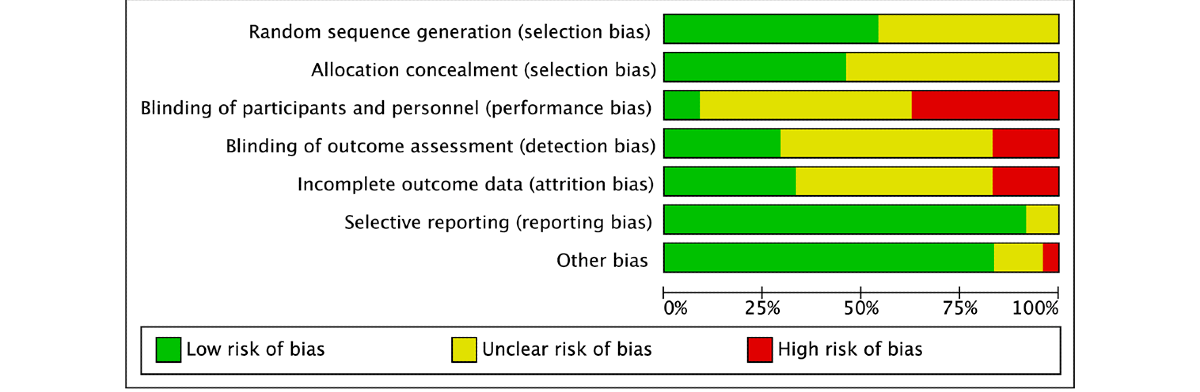
Results of the risk of bias analysis.

The overall risk of bias was relatively high, because no study was absolutely free of bias. Eight articles were rated as low risk for selection bias [30,35-38,48,53]. Others were judged to be unclear as the procedure of the sequence generation was not classified [31-34,38-46,50-52]. A total of 13 articles did not describe the methods of random allocation [31,33,38-41,43-45,47,50-52]. The risk of detection bias was high in 4 articles [37,38,43,46], as these studies were unmasked to outcome assessors; 13 studies were rated as unclear, as there was an insufficient illustration of whether they blinded the outcome investigators [32-35,41,44-48,50,52]; 2 studies were double-blind trials in which participants were blinded to treatment conditions [36,53]. The control groups consisted of sending different messages compared with the intervention groups. Four articles reported no missing data from the baseline to the endpoint [47,49,51,52]. Low risk of attrition bias was found in 8 studies [43,44,46,47,49,51-53]. They reported that less than 5% of participants withdrew from the follow-up were analyses on an intention-to-treat basis for the missing data. Four studies had a high risk of attrition bias as the rate of dropout was over 15% in each study [30,33,40,48]. 92% (22/24) of the total studies were defined as low risk of reporting bias because all outcomes included in the protocol were reported in the results [30-39,41-43,45-53]. Missing pre-specified outcomes occurred in 2 articles resulting poor clarity in reporting bias [40,44]. In addition, funding bias was considered. Two articles mentioned that OMRON, which makes sphygmomanometers, including for home use, donated the BP device [31,49]. This was identified as a funding bias. Figure 5 describes the total risk of bias in the 24 studies. The funnel plot of the comparison of SBP and DBP did not show any extreme asymmetry and outliers, which suggests no significant publication bias.

### Quality

According to the essential checklist of mHealth, a total of 16 items should be reported in the articles. The details of each item are demonstrated in Table 2. An average of 55% (9/16) of the 16 items was mentioned in each study, from the lowest to the highest proportion, 25% (4/16) and 81% (13/16), respectively. Among the essential items, technology platform, intervention delivery, and intervention content were reported in all 24 articles. Only 6 articles introduced the availability of infrastructure, which can support technology operations in the study site [30,31,34,41,43,47]. The rate of reporting of cost assessment was also low (6/24, 25%). The usability of the content testing, communication, and the technical solution to meet the target population were described in 8 studies [35,38,39,41,44,45,47,50], as well as the reporting rate of user feedback [30,31,35,37,38,42,47,50].

**Table 2:** Rate of reporting of each item in the Mobile Health Evidence Reporting and Assessment essential criteria checklist (N=24).

Items	Report rate, n (%)
Infrastructure	6 (25)
Technology platform	24 (100)
Interoperability	11 (46)
Intervention delivery	24 (100)
Intervention content	24 (100)
Content testing	8 (33)
User feedback	8 (33)
Access of individual participants	11 (46)
Cost assessment	6 (25)
Adopting input	11 (46)
Limitation for delivery at scale	13 (54)
Contextual adaptability	5 (23)
Replicability	18 (75)
Data security	12 (50)
Compliance with guideline	14 (58)
Fidelity of the intervention	10 (42)

## Discussion

### Summary of Principal Findings

This systematic review found 24 RCTs with 8933 adult patients with hypertension, which met the criteria to assess the effectiveness of mHealth-enabled interventions in supporting self-management. According to this meta-analysis, mHealth interventions resulted in better BP control, with a significant decrease of SBP and DBP by 3.78 mm Hg and 2.19 mm Hg, respectively, compared with usual care. All 24 studies showed a greater decrease in mHealth intervention groups than the control groups. Findings of this review confirmed that self-management education through mHealth was effective in increasing patients’ knowledge of hypertension and a healthy lifestyle, medication management, and self-efficacy.

Outcomes of the economic evaluations were inconsistent across the studies. A total of 2 articles reported the negative outcomes of cost focused on direct costs [37,42]. The cost of mobile technology was shown as relatively high in rural areas. In contrast, the cost of health professionals’ time in consulting in urban areas was higher than that in the rural. Thus, cost became an inevitable element when considering barriers and facilitators.

### Mobile Health Intervention Design

All interventions were conducted via mobile technologies. A total of 3 elements may have contributed to the effectiveness of self-management: First, the high intensity of medication reminders. Most studies focused on medication adherence adopted weekly automated alerts and educational or motivational messages. This increased exposure to interventions, which is impossible in routine care. Brennan et al [41] conducted a comparison between the different intensities of messages and showed that a higher frequency of SMS text messages achieved better medication adherence. However, previous research has shown that reported high-dose reminders would result in response fatigue [54].

Second, user-driven designs were frequently reflected in the interventions and consisted of customized information and patient-provider loop interactions. A total of 11 studies reported 2-way communication between patients and physicians [31,32,38,41,42,46,47,49,50,52,53]. All interventions with an interactive communication loop showed significantly positive improvement in self-management behavior and BP change. These findings were consistent with the subgroup analysis in this review. Tailoring the intervention to the specific situation and readiness of patients is considered as crucial to self-management [55]. Particularly, Liu et al [36] compared the user-driven and expert-driven group behavior change. The expert-driven group showed better behavior change, perhaps because patients from the expert-driven group had more feedback, motivational commands, and support from physicians.

Finally, most of the interventions combined different functions. A total of 12 interventions had more than 2 functions [31,36-38,40,42,44,46,49,50,52,53], and 10 studies relied on SMS text messaging as their main method, while they also linked the BP monitoring devices to a Web-based system [32,34,37,43-45,47,49-51]. According to this subgroup analysis, studies with multifaceted functions had a larger effect on SBP and DBP reduction than those with a single function. In conclusion, the tailored frequency of messages based on patients’ health status and readiness, two-way interactive communication, and multifaceted interventions can produce better effectiveness in the self-management of hypertension.

### Strengths and Limitations of Studies Included in This Review

Significant heterogeneity showed in the meta-analysis of SBP. The reason for this is the variation in interventions. It also affects the calculation of the overall estimate [56]. According to the sensitivity analysis, Margolis et al [42] was regarded as the main article, which influenced the heterogeneity.

The occurrence of heterogeneity highlighted the strengths and limitations of the included studies. Strengths are illustrated as follows: first, more than half (13/24, 54%) of the total studies conducted power calculations for clinical data outcomes [32-38,42,43,46,48,51,53]. Second, the description of each intervention provided clear and sufficient details, allowing a thorough understanding of the method. Finally, the reporting rate of detail in the research methods was high within all articles. Over 80% (21/26) of the items listed in the mERA methodological checklist were described in these studies. This showed significant progress compared with the studies included in the previous review [57].

Particularly, self-reporting bias of compliance is clearly a potential weakness of mHealth. It would increase the risk of recall and social desirability bias. The reliability of self-reporting depends partly on the educational, socioeconomic, and cultural background of participants [58]. However, studies included in this review attempted to reduce the self-reporting bias. A total of 17 articles used self-reporting in their studies [30-35,39-46,50,52,53]. Of the 17 articles, 12 articles took steps to test the validity of self-reporting data, including home visits for behavior checks, BP monitoring devices connected to websites, and random phone calls to check medication adherence [31,33,35,39-44,47,52,53].

Referring to the limitations, the duration of the studies included was relatively short. Only 10 studies lasted for or over 1 year [32,38,41-43,46-48,51,52]. The result of subgroup analysis according to the duration of trials found in this study was similar to a previous meta-analysis, which compared digital interventions with conventional methods [59]. The overall effects of SBP and DBP were inconsistent between studies with shorter and longer durations. Thus, more evidence is needed to confirm the long-term effect of mHealth.

Though all articles were published after 2010 when the CONSORT-EHEALTH statement for reporting of eHealth and mHealth interventions was released [60], many mHealth intervention details were still unreported. Though performance bias was a prominent weakness in mHealth intervention, it can be explained by the interactive nature of the interventions, which is difficult for participants to be blinded to their health care providers [61]. Small sample size was also prominent in the included studies, which would cause a huge difference in the estimates of the target population.

In addition, all studies were from high-middle and high-income countries. Similarly, the study sites were mostly in urban settings, which restricts the diversity of the target populations. This is despite the fact that one of the important benefits of mHealth is to allow patients to receive adequate care remotely [19]. Davidson [44] reported more considerable cost savings in the mHealth group than in the control group in the study of underserved populations.

The relatively homogeneous populations limited the generalizability of the mHealth intervention. It is also important to consider culture-related differences, racial diversity, and the heterogenetic patterns of mHealth interventions, which have been mentioned in discussion of almost all articles. Nevertheless, only 5 studies have examined the potential cultural adaptation of mHealth in different settings [31,32,35,41,50]. Specifying cultural and contextual adaptabilities of mHealth interventions would help clarify whether the study design can be considered as a potentially useful platform for future research. Other observable limitations include the fact that only 6 articles reported users’ satisfaction [31,32,35,37,47,50].

In relation to economic evaluations, mHealth showed only a small short-term economic benefit, but enormous potential in the longer term [62]. However, the longest duration of studies in this review is 18 months [46,52].

### Strengths and Weaknesses of This Systematic Review

To our knowledge, this is the first systematic review that analyzed the relationship between the characteristics of mHealth-enabled hypertension self-management and the clinical and behavioral outcomes, using both meta-analysis and narrative synthesis. More importantly, this review adds to a body of knowledge of the strengths and limitations of included studies against the mERA checklist.

The chief weakness is the observed heterogeneities in relation to the intervention and control features. In addition, this review only recruited RCTs and excluded other designs with analyses that might also have overcome confounding. The language was restricted to English, which reduces the diversity of studies analyzed. Moreover, the sensitivity analysis was only conducted by excluding each trial sequentially to determine the influence of a single study. Owing to the small number of studies included, studies were not divided into different categories for further sensitivity analyses.

### Implication of Policy Making and Further Research

Considering that at least one-third of patients with hypertension have uncontrolled BP, this review provided evidence that mHealth self-management could improve hypertension management and reduce the risks of stroke and CVD. There is increasing interest comparing benefits of mHealth approaches. Questions remain to be addressed about the values of diverse mHealth methods. To promote mHealth interventions of self-management effectively and efficiently, more clinical studies are warranted to detect the relationship between the specific intervention pattern and outcomes. In addition, patients’ compliance with self-management interventions should be examined in the future.

According to the generalizability, there is a necessity to determine whether mHealth-based self-management methods should be tailored to age groups, cultural contexts, or need to be extended to include support from health care personnel. Therefore, training physicians to ensure that patients’ behaviors are maintained and adopted convincingly is also necessary. Clinical trials are called for to fill the gap of techniques of appropriate combination of mHealth intervention and routine care. Thus, more long-term economic evaluation needs to be done.

### Conclusions

The intent of this systematic review was to identify and evaluate the effectiveness of mHealth-enabled self-management of hypertension from RCTs. This review clearly demonstrated that an mHealth-enabled hypertension self-management intervention was effective in improving SBP, DBP, and BP control. Both medication adherence and self-management behavior showed positive changes after the intervention. Economic evaluations presented potential cost saving in long-term effectiveness. It is the first analysis that combines clinical data and intervention features.

In conclusion, mHealth self-management has proved to be a potentially useful intervention strategy for BP management. mHealth interventions could be beneficial for BP control at the individual level and in reducing the burden of hypertension at the population level. The development of mobile technologies is especially useful when health care resources are inadequate. The broader utilization of mHealth self-management will be an important contributor to improving the quality of health care and meeting the target of universal health coverage.

## Appendix

Multimedia Appendix 1 Characteristics and designs of included studies.

Multimedia Appendix 2 Outcomes of included studies.

## Abbreviations

BP: blood pressure
CONSORT-EHEALTH: Consolidated Standards of Reporting Trials of Electronic and Mobile HEalth Applications and onLine TeleHealth
CVD: cardiovascular disease
DBP: diastolic blood pressure
LMIC: low- and middle-income country
MD: mean difference
mERA: Mobile Health Evidence Reporting and Assessment
mHealth: mobile health
OR: odds ratio
RCT: randomized controlled trial
SBP: systolic blood pressure
TAU: treatment as usual
WHO: World Health Organization
