# Supplementary material for: The Effectiveness of Self-Management of Hypertension in Adults Using Mobile Health: Systematic Review and Meta-Analysis
Source: JMIR Mhealth Uhealth. 2020 Mar 27;8(3):e17776. doi: 10.2196/17776 (PMC7148553; doi:10.2196/17776)
Supplement: Multimedia Appendix 1 [file mhealth_v8i3e17776_app1.doc]

**Multimedia Appendix 1.** Characteristics and designs of included studies (N=24).

| Study reference | Setting | Total sample size (n) | Population | Follow-up duration | Intervention content | Control content | Outcome measures |
| --- | --- | --- | --- | --- | --- | --- | --- |
| Migneault et al 2012 [35] | United States | 337 | African American, aged >34 years | 8 min | - Behavioral intervention and usual care | Usual care | - Primary: change in behavior, medication adherence - Secondary: change in BP^a^ |
| Liu et al 2018 [36] | Canada | 128 | Stage 1 or 2 hypertension, aged 35 to 74 years | 4 min | - Intervention 1: user-driven e-counseling^d^ - Intervention 2: Expert-driven e-counseling | Weekly email newsletter | - Primary: SBP^b^ - Secondary: DBP^c^, behavior |
| Nolan et al 2018 [48] | Canada | 264 | Stage 1 or 2 hypertension, aged 35 to 74 years | 12 min | - E-counseling intervention | Self-monitoring and basic self-management education | - Primary: decrease of SBP, DBP - Secondary: other clinical data |
| Contreras et al 2019 [51] | Spain | 148 | Stage 1 or 2 hypertension | 12 min | - Self-monitoring and pharmacological support | Usual care | - Primary: medication adherence - Secondary: change of SBP and DBP |
| Moore et al 2014 [47] | United States | 44 | Receiving ≤1 medication | 12 min | - Technology-supported health coach | Usual care | - Primary: decrease in SBP and DBP; the proportion of BP control - Secondary: the change in medication load and weight |
| McKinstry et al 2013 [37] | United Kingdom | 401 | Adult patient with hypertension | 6 min | - Self-monitoring and closed loop feedback | Usual care | - Primary: mean SBP - Secondary: mean DBP |
| Nolan et al 2012 [30] | Canada | 387 | Stage 1 or 2 hypertension, aged 45 to 74 years | 4 min | - E-counseling intervention | Usual care | - Primary: change of SBP and DBP |
| McManus et al 2010 [38] | United Kingdom | 480 | Receiving ≤2 antihypertensive drugs, aged 35 to 85 years | 12 min | - Self-monitoring and teleconnection with doctors | Usual care | - Primary: change in mean SBP - Secondary: antihypertensive drugs prescribed |
| Morawski et al 2018 [39] | United States | 412 | Aged 18 to 75 years | 3 min | - Medication adherence intervention | Usual care | - Primary: medication adherence and change of SBP - Secondary: the proportion of controlled BP |
| Lee et al 2016 [40] | Taiwan (China) | 382 | Aged 18 to 85 years | 6 min | - Self-monitoring and reminder intervention | Recording BP monitoring outcome and weekly reminder | - Primary: change of BP - Secondary: the degree of BP control |
| Ghezeljeh et al 2018 [53] | Iran | 100 | Aged 35 to 80 years | 1.5 min | - Intervention 1: self-management without follow-up - Intervention 2: telephone follow-up - Intervention 3: smartphone-based social networking follow-up | Routine education | - Self-management behavior |
| Brennan et al 2010 [41] | United States | 638 | African American | 12 min | - DMP^e^ with nurse support and usual care | LSP^f^ and usual care | - Primary: SBP and DBP - Secondary: frequency of BP monitoring; health care utilization |
| Margolis et al 2013 [42] | United States | 450 | Uncontrolled BP | 12 min | - Self-monitoring and phone visit of pharmacists | Usual care | - Primary: proportion of patients with controlled BP - Secondary: change in SBP and DBP; patient satisfaction |
| KIM 2019 [49] | Korea | 124 | Aged >65 years | 2 min | - Intervention 1: home-based health coaching - Intervention 2: information providing - Intervention 3: information providing and coaching | Usual care | - Primary: self-management behavior; hypertension-related knowledge - Secondary: change in SBP and DBP |
| McManus et al 2018 [43] | United Kingdom | 1182 | Aged >35 years, taking ≤3 antihypertensive medicines | 12 min | - Intervention 1: telemonitoring and send readings - Intervention 2: self-monitoring and record BP on paper | Usual care | - Primary: change of SBP - Secondary: self-reported adherence |
| Davidson et al 2015 [44] | United States | 38 | Hispanic or Latino or African American or black, aged 21 to 65 years with uncontrolled BP | 6 min | - Medication adherence and BP monitoring | Usual care | - Primary: proportion of SBP control - Secondary: proportion of DBP control; the total change of BP |
| Meurer et al 2019 [45] | United States | 55 | Emergency department with a systolic stage 2 or more hypertension | 4 min | - Medication and health behavior intervention | Usual care | - Primary: proportion of BP control - Secondary: change in SBP |
| Bosworth et al 2011 [46] | United States | 591 | Uncontrolled BP | 18 min | - Intervention 1: self-monitoring nurse-administered behavioral management and usual care - Intervention 2: self-monitoring nurse-administered physician-directed management with clinical decision support system and usual care - Intervention 3: combined 1 and 2 | Usual care | - Primary: BP control - Secondary: change of SBP and DBP |
| Maciejewski et al 2013 [52] | United States | 591 | Uncontrolled BP | 18 min | - Intervention 1: self-monitoring nurse-administered behavioral management and usual care - Intervention 2: self-monitoring nurse-administered physician-directed management with clinical decision support system and usual care - Intervention 3: combined 1 and 2 | Usual care | - Primary: BP control - Secondary: change of SBP and DBP |
| Piette et al 2012 [31] | Honduras and Mexico | 200 | Aged 18 to 80 years | 1.5 min | - Self-monitoring and structured email alerts and family members’ help | Usual care | - Primary: SBP - Secondary: depressive symptoms, medication-related problems, satisfaction |
| Chandler et al 2019 [50] | United States | 54 | Hispanic or Latino diagnosed, aged 21 to 65 years | 9 min | - Self-monitoring and electronic medication tray | ESC^g^ | - Primary: change in SBP - Secondary: DBP and adherence |
| Bove et al 2013 [33] | United States | 241 | SBP of 140 mm Hg or above | 6 min | - BP education and monitoring and usual care | Usual care | - Primary: the proportion of BP control; changes in BP - Secondary: BMI^h^ |
| Bobrow et al 2015 [32] | South Africa | 1372 | Receiving antihypertensive medication, aged ≥21 years | 12 min | - Intervention 1: information-only adherence support - Intervention 2: interactive support | Usual care | - Primary: change in mean SBP - Secondary: proportion of BP control and health status |
| Varleta et al 2017 [34] | Chile | 314 | Aged 30 to 80 years | 6 min | - Education and usual care | Usual care | - Primary: antihypertensive therapy adherence - Secondary: change in SBP and DBP |

References cited in this table: [30-53]

^a^BP: blood pressure.

^b^SBP: systolic blood pressure.

^c^DBP: diastolic blood pressure.

^d^E-counseling: electronic counseling.

^e^DMP: disease management program.

^f^LSP: light support education program.

^g^ESC: enhanced standard care.

^h^BMI: body mass index.
