# Supplementary material for: The Effectiveness of Self-Management of Hypertension in Adults Using Mobile Health: Systematic Review and Meta-Analysis
Source: JMIR Mhealth Uhealth. 2020 Mar 27;8(3):e17776. doi: 10.2196/17776 (PMC7148553; doi:10.2196/17776)
Supplement: Multimedia Appendix 2 [file mhealth_v8i3e17776_app2.doc]

**Multimedia Appendix 2.** Outcomes of included studies (N=24).

| Study reference | Blood pressure outcome | Self-management behavior outcome | Medication adherence outcome | Costs |
| --- | --- | --- | --- | --- |
| Migneault et al 2012 [35] | Larger reduction of SBP^a^ and DBP^b^ in the intervention group than the control group | Dietary: significant improvement in the intervention group (*P*=.02); activity: significantly (*P*=.02) | Not significant (*P*=.25). Higher medication adherence in the intervention group than in the control group | NR^c^ |
| Liu et al 2018 [36] | Significant for SBP (*P*<.001). A greater reduction of SBP in the expert-driven group. Not significant difference for DBP (*P*=.07) | Significant. A greater improvement of daily steps and fruit consumption in the expert-driven group (*P*=.01) | NR | NR |
|
| Nolan et al 2018 [48] | Significant. A greater reduction of SBP for e-counseling^d^ (*P*=.02). No significant difference in DBP between e-counseling versus control (*P*=.17) | NR | NR | NR |
|
| Contreras et al 2019 [51] | Significant. SBP (*P*<.001) and DBP (*P*<.001) in the intervention group are lower than in the control group | NR | Significant. Medication adherence is 86.3% in the intervention group and 62.66% in the control group (*P*<.001) | NR |
|
| Moore et al 2014 [47] | Significant difference in SBP change (*P*=.009). No significant difference in DBP change (*P*=.054). All of the participants achieved BP^e^ control | NR | NR | Intervention: US $67.50 per patient/year; Control: US $248 per patient/year |
| McKinstry et al 2013 [37] | Significant reduction of SBP (*P*<.001) and DBP (*P*=.002) in the telemonitoring group than the control group | No significant difference in lifestyle adjustment between groups (*P*=.79) | Better medication adherence in the intervention group | Costs in the intervention group is higher than in the control group by US $173.41 |
|
| Nolan et al 2012 [30] | Significant. Lower SBP pressure in the e-counseling group with 1-7 emails (*P*=.03); Significant. DBP differed between 3 e-counseling groups (*P*=.02) | NR | NR | NR |
| McManus et al 2010 [38] | Significant difference of SBP (*P*=.002). Reduction of SBP in the intervention group than in the control group by 5.4 mm Hg; No significant difference in DBP change between the groups (*P*=.09) | Quality of life increased in the intervention group | NR | NR |
| Morawski et al 2018 [39] | No difference in DBP change between the groups (*P*=.78) | NR | Significant. Higher in the intervention than control (*P*=.01) | NR |
|
| Lee et al 2016 [40] | Significant. Difference in SBP change between 2 groups by 0.69 mm Hg (*P*=.04); No significant difference in DBP (*P*=.35) | NR | NR | NR |
| Ghezeljeh et al 2018 [53] | NR | Significant. Better self-management behavior in intervention groups than control (*P*<.001) | NR | NR |
| Brennan et al 2010 [41] | Significant. Lower SBP of the intervention group than the control group (123.6 vs 126.7 mm Hg; *P*=.03) | Significant. The intervention group is 46% more willing to report weekly BP monitoring than the control group (*P*=.02) | Significant. Better medication adherence in the intervention than the control group (*P*=.01) | NR |
|
| Margolis et al 2013 [42] | Significant. Difference of SBP change between intervention and control group: −9.7 mm Hg (*P*<.001); Significant. Difference of DBP change between intervention and control group: −5.1 mmHg (*P*<.001) | NR | Difference between groups of self-reported medication adherence: 13.8% | Intervention cost US $1350 per patient |
| Kim 2019 [49] | Significant. More reduction of SBP (*P*<.001) and DBP (*P*=.02) in 3 intervention groups than the control group | Significant differences in self-management behavior (*P*<.001)，hypertension knowledge (*P*<.001), between 4 groups | Significant. Better medication adherence in the coaching group than the other 3 groups (*P*<.001) | NR |
|
| McManus et al 2018 [43] | Significant. Lower SBP in telemonitoring group than in the control group (*P*<.001); No significant difference between the 2 intervention groups (*P*=.18) | No significant difference in self-reported adherence between 3 groups (*P*=.83) | NR | NR |
| Davidson et al 2015 [44] | Significant for SBP control (*P*=.003) and DBP control (*P*=.04). Higher proportion of SBP and DBP control in intervention group than in the control group | NR | Higher medication adherence in the intervention group than in the control | Overall cost savings of US $23,692 in the intervention group; US $5,923 in the control group |
| Meurer et al 2019 [45] | SBP of the intervention group had a mean drop of 9.1 mm Hg | NR | NR | NR |
|
| Bosworth et al 2011 [46] | Significant difference in the rate of BP control in the 2 intervention groups relative to the control group (*P*=.03) | NR | NR | US $947 for behavioral management; US $1275 for medication management; US $1153 for the combined intervention arm |
| Maciejewski et al 2013 [52] | Significant (*P*<.001). Behavioral group (17.1%), medication management group (20.2%), and the combined group (20.4%) had greater SBP control compared with usual care | NR | NR | Estimate expenditures are similar |
| Piette et al 2012 [31] | Significant. 57% of intervention, 38% of the control group had controlled BP (*P* = .006); No significant decrease in SBP among intervention and control group (*P*=.74) | NR | NR | NR |
| Chandler et al 2019 [50] | Significant difference for SBP control (*P*=.009); No significant difference of DBP change (*P*=.34) showed in the intervention group and the control group | Better protocol adherence showed in the intervention group | Significant. Greater increases in intervention group than the control group (*P*<.001) | NR |
|
| Bove et al 2013 [33] | Not significant. Greater reduction in SBP (*P*=.12) and DBP (*P*=.17) in the telemedicine group than the control | NR | No significant change in the 2 groups (*P*=.86) | NR |
|
| Bobrow et al 2015 [32] | Significant (*P*=.05). The difference in SBP change of interactivity and information group compared with the control group was −2.2 mm Hg and −1.6 mm Hg | NR | Significant change between intervention and control groups (*P*<.001) | NR |
| Varleta et al 2017 [34] | Not enough power to make statistical comparisons | NR | Significant. Adherence improved in the intervention group from 49% to 62.3% (*P*=.01) | NR |
|

References cited in this table: [30-53]

^a^SBP: systolic blood pressure.

^b^DBP: diastolic blood pressure.

^c^NR: not reported.

^d^E-counseling: electronic counseling.

^e^BP: blood pressure.
